# Supplementary material for: Synergistic Modulation of Seed Metabolites and Enzymatic Antioxidants Tweaks Moisture Stress Tolerance in Non-Cultivated Traditional Rice Genotypes during Germination
Source: Plants (Basel). 2022 Mar 14;11(6):775. doi: 10.3390/plants11060775 (PMC8955497; doi:10.3390/plants11060775)
Supplement: Supplementary file 1 [file plants-11-00775-s001.zip › plants-1615883-supplementary.pdf]

Table S1. Details of collected rice landraces from different agro-ecological zones.

Agro-climatic Zone	Soil type	Landraces
North Eastern Zone	Red Sandy Loam, Clay Loam, Saline coastal Alluvium	Kar samba, Karudan samba, Karukot, Kadaikannan, Kattanoor, Krishna hemavathi, *Sadhabahar*, Kalanamak, *Kalinga-3, Jai Sree Ram, Uma*, Baskadam
North Western Zone	Non Calcareous Red, Non Calcareous Brown, Calcareous Black	Maranellu, Kalaheri, Milagi, Mollikarumbu, *Vanapraba, Virendra*, White sannam, Thuyamalli, China punchai
Cauvery Delta Zone	Red Loamy, Alluvium	Kuruvaikar, Seeraga samba, Kichadi samba, Kallinga, Kuruvaikalanchiyam, Meikuruvai, Surakuruvai, Kullakar, Kichali samba, Kattu samba, Thanga samba, Kothamalli samba, Milagu Samba, Aathur Kichadi Samba, Mapillai samba, Karuvalli, Eravipondi
Southern Zone	Coastal Alluvium , Black, Red Sandy soil, Deep red soil	Kavuni, Chandaikar, Kuliyadichan, Chithiraikar, Nootripathu, Poonkar, Mallikar, *Rajalakshmi*, Sivappumalli, Thamarai, Sivappukavuni, Norungan, AanaiKomban, Kattuyanam, Navarai, Karuppu Kavuni, *Swarna*
High Rainfall Zone	Saline Coastal, Alluvium, Deep Red Loam	Mattaikar, Adukan, *Anjali, Annada*, Mulampunchan, Abya, *Sahbagidhan*, Pokkali, *Bharathi, Jaya*, Kayumma, Kottara samba, Muttakaruva, Chenellu, Chembavu, Thondi, Aryan, Chenthadi, Kollam samba, Nochin samba, KunjuKurju, *Akshayaponni*, Chitteni, Chuvanna, Varakkuranellu, Chunjamkarnellu, ThavalaKannum, Vattan, Pattani, Ohenellu, Chakhaepoirecton, Chakhaeamubi , Eluppai poo samba, Chemban, Chumala, Kerala kandhasala, Arikiraavi, Chenkayama, Oheruchitteni, *Vandhana*, Varaputha, Veethirupa, Naatuponni, Arubathamkodai
North Eastern part of India (Meghalaya)	Loamy to fine loamy	MBR (Meghalaya black rice)

*Seed materials are collected from farmers in the respective zones. The checks IR 64 and IR64 DRT obtained from DPB &G, A.C and R.I, Killikulam.

Table S2. Proportion of variance, cumulative proportion and eigen values of rice genotypes during seed germination.

**Statistics**	**PC1**	**PC2**	**PC3**	**PC4**	**PC5**	**PC6**	**PC7**	**PC8**	**PC9**	**PC10**	**PC11**
					**DS**						
Proportion of Variance	66.61	12.13	10.19	4.73	2.60	2.10	0.81	0.57	0.14	0.08	0.02
Cumulative Proportion	66.61	78.73	88.93	93.66	96.27	98.37	99.18	99.75	99.90	99.98	100.00
EigenValues	8.66	1.58	1.33	0.62	0.34	0.28	0.11	0.07	0.02	0.01	0.00
					**NS**						
Proportion of Variance	41.87	23.93	13.06	7.74	4.98	2.79	2.39	1.70	0.96	0.47	0.06
Cumulative Proportion	41.87	65.80	78.86	86.61	91.60	94.39	96.79	98.49	99.45	99.93	100.00
EigenValues	5.44	3.11	1.69	1.00	0.64	0.36	0.31	0.22	0.12	0.06	0.01

Table S3. Contribution of different drought tolerance contributing attributes towards total variance in rice genotypes.

Attributes	**Eigen vectors**									
	PC1		PC2		PC3		PC4		PC5	
	DS	NS	DS	NS	DS	NS	DS	NS	DS	NS
Amylase	**0.94**	**0.69**	-0.13	-0.44	-0.06	0.13	-0.06	-0.47	-0.25	0.12
Protease	**0.95**	**0.85**	-0.09	**0.34**	0.02	-0.21	-0.04	0.05	0.13	0.21
Lipase	**0.96**	**0.96**	0.03	-0.02	0.17	-0.04	0.07	-0.01	0.03	0.13
Amino acids	-0.11	0.06	**0.91**	**0.46**	-0.08	-0.69	**0.33**	**0.45**	-0.06	0.04
Starch	-0.87	-0.37	0.09	**0.31**	0.24	**0.75**	0.30	0.05	0.06	-0.09
Protein	-0.88	-0.35	0.12	-0.72	-0.35	-0.02	0.07	0.26	0.24	0.31
TSS	**0.96**	**0.93**	0.18	-0.18	0.12	0.06	0.09	-0.19	-0.03	0.08
GA	**0.96**	**0.96**	-0.03	-0.04	0.10	-0.07	0.14	0.06	0.08	-0.13
IAA	**0.56**	0.16	-0.40	-0.84	-0.52	0.03	**0.42**	0.12	0.08	-0.43
CAT	**0.78**	**0.44**	0.32	**0.72**	0.18	0.23	-0.24	0.13	**0.38**	-0.31
SOD	-0.61	-0.17	0.02	**0.82**	**0.72**	0.04	0.04	-0.38	-0.04	0.11
Proline	**0.83**	**0.92**	-0.11	0.03	**0.39**	0.14	0.31	0.21	-0.00	-0.16
RWC	**0.68**	**0.33**	**0.60**	0.03	-0.29	**0.69**	-0.15	**0.47**	-0.14	**0.33**

Table S4. Correlation coefficients among various attributes under controlled environment (C) during seed germination in selected rice genotypes.

	GR	Amylase	Protease	Lipase	AA	Starch	Protein	TSS	GA	IAA	CAT	SOD	Proline
Amylase	0.399												
Protease	0.767**	0.379											
Lipase	0.693**	0.709**	0.843**										
AA	0.226	-0.419	0.351	0.123									
Starch	-0.607	-0.312	-0.377	-0.365	-0.311								
Protein	-0.517	0.007	-0.500	-0.279	-0.210	-0.129							
TSS	0.635*	0.827**	0.735**	0.908**	-0.155	-0.365	-0.228						
GA	0.632*	0.654*	0.798**	0.878**	0.107	-0.425	-0.336	0.875**					
IAA	-0.115	0.376	-0.266	0.147	-0.350	-0.284	0.451	0.247	0.228				
CAT	0.414	-0.079	0.498*	0.310	0.215	0.201	-0.638*	0.244	0.456	-0.387			
SOD	0.266	-0.262	0.067	-0.146	0.190	0.274	-0.525	-0.239	-0.282	-0.759**	0.485		
Proline	0.584*	0.534*	0.744**	0.898**	0.076	-0.176	-0.317	0.791**	0.888**	0.237	0.550	-0.181	
RWC	-0.097	0.118	0.224	0.301	-0.230	0.323	0.008	0.281	0.255	-0.008	0.293	-0.118	0.429

Table S5. Correlation coefficients among various attributes under induced moisture stress during seed germination in selected rice genotypes.

	GR	Amylase	Protease	Lipase	AA	Starch	Protein	TSS	GA	IAA	CAT	SOD	Proline
Amylase	0.627*												
Protease	0.531*	0.855**											
Lipase	0.512*	0.878**	0.914**										
AA	0.178	-0.218	-0.225	-0.082									
Starch	-0.796**	-0.908**	-0.844**	-0.764**	0.227								
Protein	0.565*	0.877**	0.895**	0.973**	0.074	-0.755**	-0.876**						
TSS	0.489	0.870**	0.927**	0.971**	-0.095	-0.786**	-0.853**	0.932**					
GA	0.431	0.594*	0.555*	0.457	-0.230	-0.569*	-0.328	0.444	0.552				
IAA	0.620*	0.625*	0.771**	0.793**	0.103	-0.683**	-0.657*	0.813**	0.753**	0.178			
CAT	-0.712**	-0.599	-0.570*	-0.499*	0.085	0.670**	0.279	-0.499*	-0.532	-0.653*	-0.345		
SOD	0.136	0.740**	0.816**	0.909**	-0.147	-0.516*	-0.871**	0.872**	0.892**	0.413	0.603*	-0.267	
Proline	0.790**	0.602*	0.607*	0.617*	0.436	-0.637*	-0.468	0.731**	0.558	0.212	0.645*	-0.638*	0.362
RWC	0.565*	0.877**	0.895**	0.973**	0.074	-0.755**	-0.876**						

Table S6. Comparison of Cytokinin and ABA content during seed germination.

Genotypes	Cytokinin (ug.g^{-1})		Per cent reduction over control	ABA (ug.g^{-1})		Per cent Increase over control
	-1.5 MPa	Control		-1.5 MPa	Control	
Kuliyadichan	0.37	0.43	13[g]	0.18	0.11	38.4[a]
Rajalakshmi	0.27	0.37	29[b]	0.12	0.09	33.3[c]
Sahbhagidhan	0.31	0.39	21[d]	0.14	0.08	27.3[e]
Chandaikar	0.28	0.35	19[e]	0.13	0.09	30.0[d]
Mallikar	0.23	0.31	25[c]	0.11	0.07	37.5[b]
IR64 Drt1	0.33	0.40	18[f]	0.12	0.08	33.3[c]
IR 64	0.09	0.24	63[a]	0.07	0.05	16.7[f]

Values are mean values of three replications followed by the same letter in each column are not significantly different from each other as determined by DMRT (p <0.05).

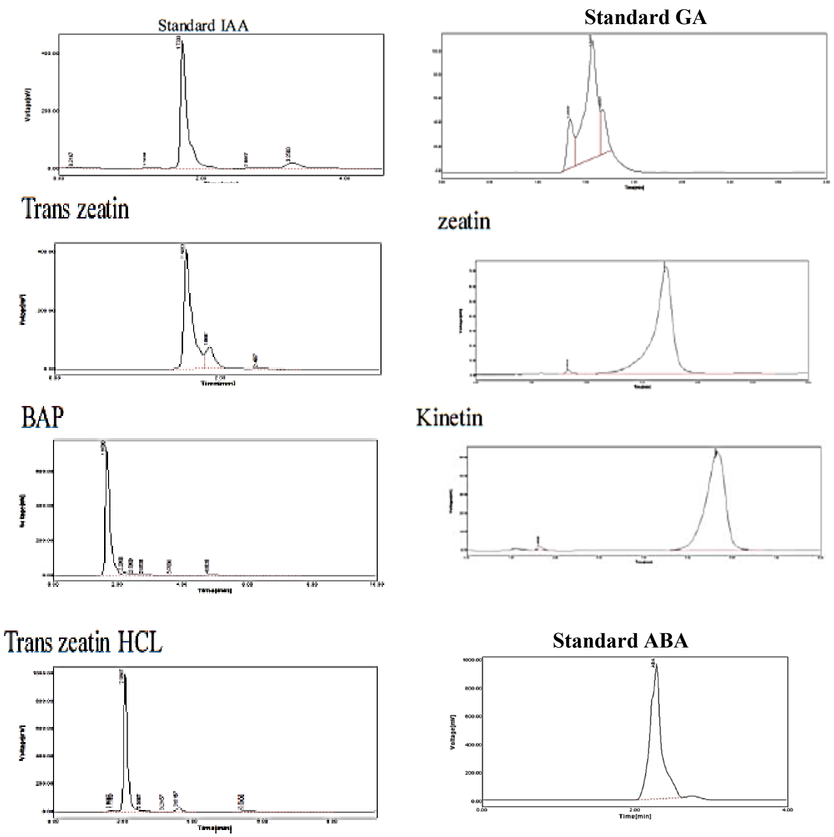

Figure S1. HPLC chromatogram for standard phytohormones IAA, GA, Cytokinin and ABA at 100 ppm.

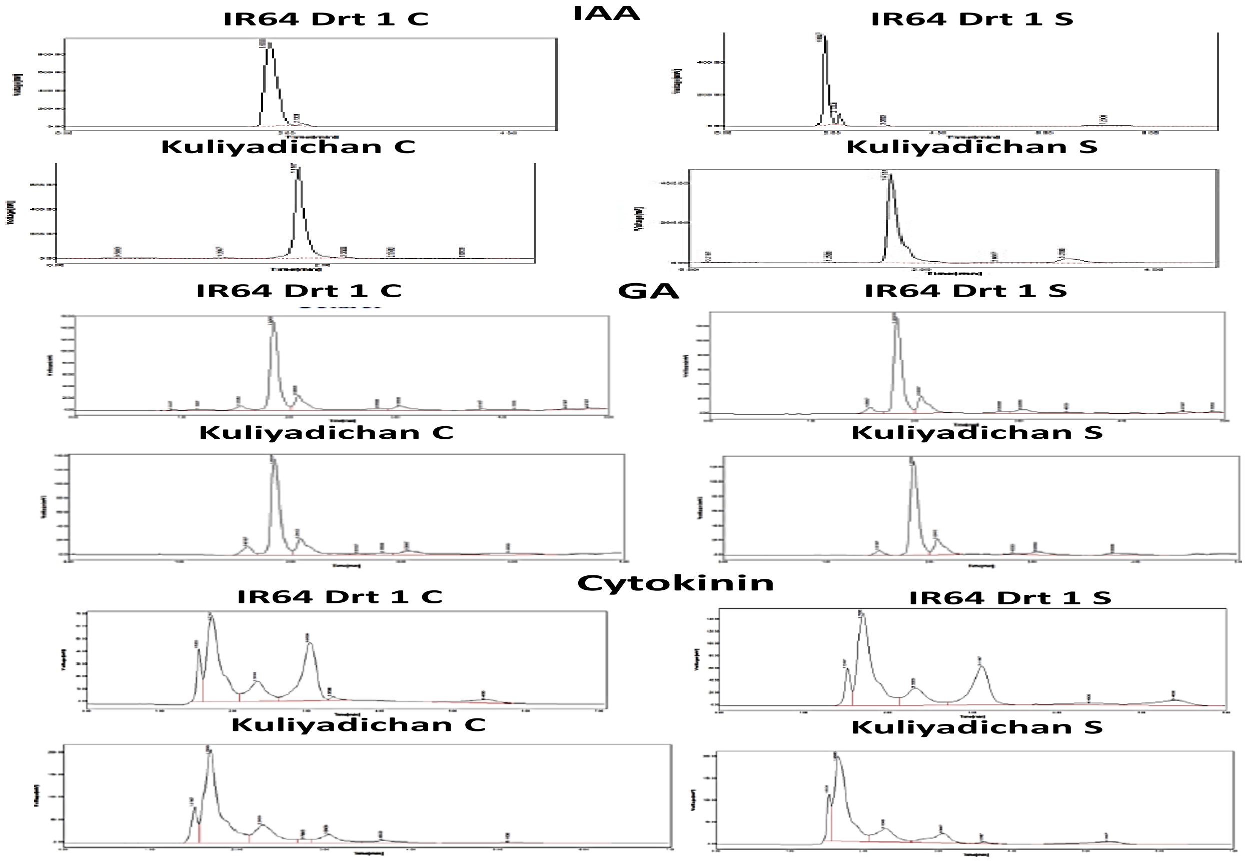

Figure S2. HPLC chromatograms depicting phytohormones for the promising genotype Kuliyadichan in comparison with IR64 (Drt1). (HPLC chromatograms depicting phytohormones for the promising genotype Kuliyadichan in comparison with IR64(Drt1). The standard chromatograms are given in supplementary material S7. .a) S- induced moisture stress of -1.5MPa. b) C- controlled environment.
